# Factors influencing the use of reproductive health care services among married adolescent girls in Dang District, Nepal: a qualitative study

**DOI:** 10.1186/s12884-019-2298-3

**Published:** 2019-05-03

**Authors:** Binita Maharjan, Poonam Rishal, Joar Svanemyr

**Affiliations:** 1 International Community Health, Department of Community Medicine and Global Health, University of Oslo, P.O. Box 1130, Blindern, 0318 Oslo, Norway; 2 Institute of Public Health and General Practice, Norwegian University of Science and Technology, P.O Box 8905, 7491 Trondheim, Norway; 3 Michelsen Institute and University of Bergen, Bergen, Norway; 4 Chr. Michelsen Institute, P.O. Box 6033, Bedriftssenteret, 5892 Bergen, Norway

**Keywords:** Child marriage, Reproductive health, Health care services, Health-seeking behaviour, And adolescent health

## Abstract

**Background:**

Child marriage is associated with adverse reproductive health outcomes, and the practice is still alarmingly common. Together with efforts to end child marriage, it is essential to provide adequate health care to already married adolescents. However, to date there has been very limited research on health care-seeking practices among married adolescents in Nepal.

**Method:**

The study was conducted in a rural part of Dang District situated in the Mid-western region of Nepal. We combined thirteen individual interviews and four focus group discussions with 17–20 years old women who had married before the age of 18 years and individual interviews with 10 key informants.

**Results:**

Pressure to give birth early, limited autonomy, and little knowledge about reproductive health issues make married adolescents vulnerable to risky pregnancies. Early-married women face a range of barriers to use existing health services including work overload, transport and distance to health care facilities, qualities of services, verbal abuse by health care providers, and shyness and embarrassment.

**Conclusion:**

Women who marry and become pregnant during adolescence face a number of barriers that limit their access to health care services and they need more attention from the health services and policy makers. More youth friendly health services and education about sexual and reproductive health should be key elements in strategies to address the health issues of early-married women and adolescent girls.

## Plain English summary

Child marriage is still common in many countries, including in Nepal where approximately half of all women aged between 20 and 49 were married before they were 18 years old. Child marriage is often associated with poor health among them and the newborns. This qualitative study examines perceptions of health and use of health services among early-married women who had given birth. We found that a combination of pressure to give birth early, limited autonomy, and little knowledge about reproductive health issues makes young married girls vulnerable to risky pregnancies. Early-married women face a range of barriers against using existing health services including work overload, transport and distance to health care facilities, qualities of services, verbal abuse by health care providers, and shyness and embarrassment. More youth-friendly health services and education about sexual and reproductive health should be key elements in the strategies to address the health issues of early-married women and adolescent girls.

## Background

Nepal ranks among the twenty countries in the world with the highest rates of child marriage [1]. In 2014, 52% of women in Nepal aged 20–49 years had married as children, and 17% of them married before the age of 15 years [2]. However, the rates are slowly declining. Among women 20 to 24 years old, 37% had married before they were 18 years old, and 10% of women from the same age range had married before they were 15 years old [3]. This indicates that Nepal has made progress in preventing child marriage, but there is still a long way to go before child marriage is eradicated.

Married girls are expected to get pregnant immediately after marriage and are at a higher risk of maternal and child morbidity and mortality, compared to women married at a later age [1, 2, 4, 5]. It should be noted that the great majority of those who get pregnant at an early age are married [6].

Globally, complications during pregnancy and childbirth is the second most common cause of death for adolescent girls [7]. Annually, approximately 70,000 maternal deaths in low and middle-income countries are associated with adolescent pregnancies [8]. Girls younger than 15 or 16 years have increased risks of morbidity and mortality compared with older adolescents [4, 9].

Child marriage is associated with numerous health consequences**.** Married girls have a higher risk for mental illness and are more likely to experience physical and sexual violence at the hands of their spouses as compared to unmarried ones [5, 10]. They also have limited opportunity to continue their education or find employment and do not participate in domestic decision-making. Evidence from India indicates that younger age at marriage is associated with increased socio-economic vulnerabilities and greater gender inequities, along with a lower likelihood of antenatal care and contraceptive use [5]. Findings from India also reveal that, compared to women who marry before age 18, women who marry later are more likely to be involved in planning marriage, to reject wife beating, to use contraceptives to delay their first pregnancy and to have their first birth in a health facility [11].

Along with implementing strategies and efforts to prevent child marriage globally, it is necessary to strengthen the support to married adolescents [5]. Married adolescent girls’ needs for adequate health care services are widely recognised but under-researched [12]. In Nepal, only a few studies have addressed health-seeking behaviour of married girls and young women. Thus, the primary aim of this study is to explore and increase knowledge on health care-seeking practices among married adolescent girls in Nepal. The secondary aim is to explore facilitators and barriers to reproductive health care utilisation among young mothers in rural villages of Dang District, Nepal.

## Methods

### Study design

This study was an exploratory qualitative study using a combination of individual semi-structured interviews with early-married females and key informants and Focus Group Discussions (FGD) with early-married females. Triangulation of methods adds credibility to the study as it provides different perspectives on the same phenomenon [13]. It also minimises bias that may relate to the position of the researcher [14].

### Study sites

Nepal is divided into five development regions, which are further divided into 14 zones, 75 districts, 58 municipalities, 3915 village development committees (VDCs) and approximately 3600 wards. Municipalities are usually urban areas while VDCs are rural areas [15]. The study was conducted in Dang District, situated in the Mid-western region of Nepal. It has 31 VDCs and four municipalities. Dang was purposively selected as a study site because data from the region showed a high prevalence of child marriage [16]. Four neighbouring VDCs in rural areas, Hekuli, Shreegaun, Dhanuri and Urahari, were selected based on the information provided by a local non-governmental organisation (NGO) and because these were accessible by public transport. Male members of the community were mostly engaged in small-scale farming and agriculture, labour work, or worked abroad. Married women were mostly engaged in household work and farming. Staple foods of the region are rice, maize and locally grown vegetables. All VDCs have one health post and a primary health facility where only basic health care is provided within 30–60 min walking distance of the village. Most inhabitants of these VDCs are from the marginalised ‘Tharus’ and ‘Chaudhary’ groups. The villages have electricity, but power outages are common. They have access to water from wells, but no practice of purifying drinking water.

### Data collection

In-depth individual interviews (IDI) were held with thirteen girls/women who were married before the age of 18 years and had at least one child or were pregnant at the time of the interview. One participant who neither had a child nor was pregnant was included in the study because she had had a miscarriage and married as a child, therefore her experiences were still relevant. Participants were aged between 17 and 20 years old. We contacted participants with the help of social mobilisers working for NGOs in the particular VDCs. In addition, through snowball sampling, we asked research participants to provide information about other women in the same category who could be willing to participate [17].

We organised four focus group discussions (FGDs) with the same category of girls and women in each of the VDCs and with six to eight girls/women in the groups. We used the same selection criteria for participants in FGDs as for the individual interviews. We included participants from different socio-demographic backgrounds in terms of caste, ethnicity, and education. FGDs lasted from 45 min to 1 h and 15 min.

We conducted individual interviews with ten key informants in the community including a school headmaster, a health post in-charge, two social mobilisers (NGO staffs), a female community health volunteer (FCHV), a head of department officer (district), a VDC officer, a community leader, a human rights officer, and an in-charge officer of NGO. These interviews lasted from 20 min to around 47 min. Interviewees and FGD participants were selected purposively. Intentional selection of participants provided a basis for collection of rich information related to the subject area of the study [18] and maximum variation in research participants [19].

The first author collected data with the help of research assistants, who were trained social workers. They were included in pilot FGDs and interviews to ensure that they understood the concept and objectives of the study and their roles in data collection. To develop trust and good working relationships between the researcher, research assistants and study participants, more than a week was spent in the villages communicating with all parties and explaining the purpose of the study [19].

An audiotape recorder was used for recording interviews and FGDs, while note taking by the research assistants was used in case audiotaping failed [20]. Data collection and transcription took approximately three months. Interviews were in Nepali language and translated into English. Transcribed data were then shared with a supervisor (second author) who could read both languages. Any discrepancies were removed and corrected in line with changes suggested by the supervisor.

### Analysis

The first author translated the initial interview scripts from Nepali to English and shared them with the second author who can read both Nepali and English. Systematic text condensation (STC) strategy, a method developed to analyse qualitative data, was used. It consists of four main steps: construction of preliminary themes by identifying associated and disassociated themes, having themes converted into codes by identified meaning units, condensation from constructed codes to meaning units following re-contextualisation of data into the formation of descriptions and concepts [21]. Transcripts were then converted into preliminary themes. Using the HyperRESEARCH software for qualitative analysis [22], meaning units were generated, from which codes were developed further into code groups. During the process, reflection on the categories and the interviews continued until the final categories emerged.

## Results

### Participants’ characteristics

The women participants were aged between 17 and 20 years. Husbands of all participants were one to three years older, and most of them had gone abroad (India, Qatar) to earn money to provide for their families. Mostly, we observed women working in the farming fields. All participants could read and write basic Nepali language; however, most of them had already dropped out from school. Very few participants continued school after marriage and childbirth.

### Living conditions and perceptions of health and illness

The primary role of the women in Dang District is to remain in the house and perform household tasks. An overload of domestic work, which includes carrying grass for cattle, water, barley sacks and others, prevented the women from caring for their health during pregnancy. Being young, they have very little negotiating power and must work hard to meet their families’ expectations. One participant said:

*“Doctors told me not do the heavy lifting, not to carry the heavy water but I used to carry it. There was nobody at home, my mother-in-law was always quarrelling with my husband, insisting on me working.” (Married at 15 years, present age 19 -20 years, IDI).*

This expression corresponded with the views of the key informants:

*“They have to finish all household work before they can visit the health-post, like handling children, food and everything. After finishing household work they start to go for health-post and the time they reach there, the health posts are closed.” (District officer, local NGO)*

Most women participants talked about verbal violence inflicted by the husband and mother-in-law. They cited inability to perform the household work as reason for this violence.

*“Even I got married at very early age, I used to do all work. But even after that, they (her in-laws) would not be satisfied with me. They used to scold me.” (Married at 14 years, Present age 19–20 years)*

Severe pain was an important factor when considering the use of health care services. Some participants visited local health care facilities for problems like leg pain, hand pain, and fever, which were considered minor illnesses, while others did not consider it severe enough to seek treatment. Only when in severe pain, they would go to hospital for medicine.

With regard to labour pain, participants considered initial and mild pain as normal.

*“I started having pain, and the pain was on and off. I thought it was abdominal pain only, and I told nothing to anybody. And in the evening, I started to have severe pain.” (Married at 16 years, present age 19–20 years)*

All participants got pregnant as soon as possible after getting married. Neither the girls themselves, nor the husbands or the mothers and fathers-in-law were in favour of postponing the first pregnancy. The health of the girl was not prioritised when she was expecting a baby:

*“My husband didn't agree to use the family planning devices. My mother and father-in-law also would not agree if we were not having a baby after marriage. They insisted on me having a baby.” (Married at 14 years, present age 17–18 years)*

Participants also think that delaying the first pregnancy by a year or more is too long:

*“It was becoming more than one year since I got married so we decided to have baby … I do not feel any discomfort and it (giving birth) is not about happiness. One should have at least one baby.” (Early married, present age 18–19 years)*

Most key informants considered young pregnant women to be physically and mentally immature and as having no knowledge about how to care for themselves and the baby during pregnancy. Adolescent participants expressed similar thoughts.

### Opinions and experiences with reproductive health care services

Married women would not use contraception before the birth of the first child. Besides the urge to get pregnant, they believed that if they used modern contraception it would be difficult to become pregnant.

*“Usually, there is a risk of not having a baby after the use of family planning methods before the first pregnancy. After marriage, the thinking is that it is not necessary to use it (laughs). After the baby is born, temporary methods are used.” (Married at 17 years, present age 19–20 years, FGD3)*

However, many participants started using family planning methods because they suffered from complications during their first pregnancy and were not fully capable to care for the baby. This was also expressed in key informant interviews.

When husbands or family-in-laws did not accept use of contraception, some women decided to use these secretly:

*“I didn’t ask anybody about this, when keeping the “Norplant”[implant]. I told them, that day, I was going to my mother’s house and I went to the hospital and had the implant and I came here after it was healed.” (Married before 18 years, present age 19–20 years)*

#### Antenatal care

A common facilitator for seeking health care during pregnancy was information and suggestions provided by experienced women in the community or by relatives, especially when symptoms such as vomiting, headaches, dizziness or delayed menstruation arose.

*“I am 19 years now... I became pregnant... I started to vomit after two months of my pregnancy. And then I got to know about my pregnancy. It was “didibahini”- “elder sisters” from village told me to go for the examination” (Married at 17 years, present age 19–20 years, FGD2)*

Another factor was information and motivation received from health care professionals.

*“Social workers used to come here very often to explain about the antenatal examination. They also explained about taking the tetanus injections. Social workers came home to call me and then I used to go … When the social worker knows about the pregnancy, they come to visit at home. They come to call us.” (17 years married, present age 19–20 years)*

However, the use of antenatal care varied from one to more than four visits.

*“I completed both injections at once in antenatal care visit when I was pregnant with my daughter but with my son I went twice. That's it. I went for check-up once during first pregnancy and didn't go for an antenatal visit in my second pregnancy.” (Married at 14 years, present age 17–18 years*)

Most participants were in favour of going to hospital for delivery.

*“It is usually good to go to the hospital when giving birth. There is a risk of losing the life … that is why I feel it is good to give birth in the hospital. For me, it was absolutely good when I was giving birth.” (Married at 16 years, present age 19–20 years, FGD 3)*

Some participants had a positive perception of health care facilities.

*“I feel it is good. When I take my child to hospital, this encourages me. There is a hope that they will be able to do something for him.” (17 years married, present age 19–20 years)*

However, some participants complained about the harsh language used by health care providers. They received critical comments about their age of marriage and pregnancy, and several said that negative experiences with health workers affected their choices:

*“They had already scolded me for first pregnancy at very young age. I was furious at that time. If I had gone for the check-up for second pregnancy, they would even scold me very badly. That is why I didn't go for a check-up. They speak loudly and don’t show respect. Who would do it if one knew everything? If I had known everything, I would not be pregnant. ( … ) I feel like they are very mean and do things for their benefits. I was thinking, and I delivered my second baby at home. I didn’t go to medical centre. I was returning from my mother’s home, I delivered my daughter on the way. There was a home of a midwife nearby, I delivered my second baby there.” (Married at 14 years, present age 17–18 years)*

FGD participants also reported negative experiences:

*“The doctor shouted at me it is not only you who give birth to a baby, you elope at the age of 17 and you women are looking for your rights. The doctor was scolding me. And I was not in the position to feed the breast milk. I had wounds in my body and I was in severe pain, my body was aching.” (Married at 17 years, present age 19–20 years, FGD1)*

Verbal abuse by health care personnel in the hospital setting influenced the preferred place of delivery and increased the probability of avoiding health services. Participants described that they had visited various facilities. Mostly women went to the city area to seek treatment when they were not satisfied with local health care and when resources at local facilities were inadequate.

“( … ) *The family members took me to the health-post. I was kept in Baghmare (rural area) health post for 4 to 5 hours … . There, my baby was not born. When I was not able to give birth, I was taken to Ghorahi (city area) hospital. Then when I reached the hospital; I gave birth after almost 1 and 1/2 hours.” (Married at 17 years, present age 19–20 years, FGD2)*

One woman considered the government healthcare facilities good for childbirth, but chose private healthcare facilities for treatment for other illnesses.

*“The government one is for delivering the baby. It is usually the private ones we go to for other illness. May be the services are good in private ones.”(Married at 17 years, Present age 19–20 years)*

Some had complaints about the quality of care that did not necessarily relate to their age.

*“I felt bad when I had delivered my baby, the nurses left without doing the stitch in some area, which caused bleeding and again they repeated the stitching process (in the private part) 2 or 3 times. That has happened to me.” (Married at 15 years, present age 19–20 years)*

Other barriers related to the participants’ shyness and discomfort. A key informant explained that girls are not comfortable in seeking health care:

*“One of our students studying at grade 6 or 7 got married, and she dropped out of school. She married at a very low age and became pregnant at a very low age. They feel discomfort and are also afraid to go for examination as they think somebody might tell them that they got pregnant at a very low age, and they are shy.” (Key informant, Headmaster, Government secondary school)*

A participant confirmed that her reason for favouring home delivery was shyness and discomfort.

*“Although I learned about the need of having the examination and taking the injections at school when I was a student, I was avoiding it. There was a feeling of shyness within me.” (17 years married, present age 19–20 years)*

A key informant emphasised that female patients tend to hide reproductive health problems from male health care workers and instead mention a general pain like a headache, which is not real.

*“They go with one problem, and they say they have a headache, which is easier for them to tell and bring the wrong medicine. Older women are going to health-post easily after delivery also, but these early-married women are very shy to go.” (Key informant, In-charge local NGO)*

### Structural factors affecting choice of health care

Some of the challenges and barriers young pregnant women had to deal with were not particular for adolescents. The major barrier in the villages of Dang District was the inexistence of proper transport facilities. Common means of transport were bicycle and motorbike, which were used especially by male members of the community. Use of public transport was very rare because of unavailability. To give birth, ambulances were called mostly from the city area, but did not always come in time or was called too late. Two women shared their experiences of travelling by motorbike during labour pain.

*“We managed to travel with the help of motorbike. It could have been faster if we could get an ambulance. But we did not manage to find one, it was raining, maybe it took about one and half hour for us to reach there (hospital).” (Married at 17 years, present age 19–20 years)*

A few participants delivered at home without the assistance of skilled attendants because they did not have time to go to the clinic or hospital.

*“I also delivered the baby at home. I was working all morning … and in the evening, I delivered the baby … the labour pain was for not so long. Nobody was aware at home that I was having the labour pain.” (Married at 16 years, present age 19–20 years, FGD3)*

Coming from a minority caste and ethnicity were identified as a potential barrier to approaching health care facilities.

*“In our society, the patients are treated according to the family background. If the person is rich and has high status, then she is viewed differently. The one from a poor background like Dalits, Janajati, are treated differently. On top of that, adolescents are more vulnerable; they are humiliated and treated differently.” (Key informant, District officer local NGO)*

Participants explained that early marriage brought economic instability, which subsequently altered choice of health care and facilities. They relied on family, relatives and loans for health care.

*“Main problem is, he (husband) is not educated and he is not getting any jobs. He has some work, but that money doesn’t cover any expenses. For food it is okay, but it is difficult to cover the treatment expenses. It is difficult to take the loans.” (Married at 14 years, Present age 19–20 years)*

Although delivery is free in government health care, some experienced excessive expenses because of health complications with the baby at the time of delivery.

*“I had a lot of expenses at hospital. The expense was almost like sixty to seventy thousand rupees. For me there was not much expenses, it was all for this child.” (Married at 16 years, present age 17–18 years)*

Key informants repeatedly talked about cash benefits and incentives for antenatal examination and delivery offered by the government. Most participants, however, were not aware of it. A few of them received money from health care facilities after they gave birth to their babies in hospital but did not necessarily understand why.

*“I went there at my own expense. Later, they gave me thousand rupees after the birth of the baby. I don't know if it is for the transport expenses or for the food expenses.” (Married at 15 years, present age 19–20 years)*

Some participants spoke of difficulties in receiving incentives after childbirth in health care facilities.

*“I had the antenatal card but I had done check-up once, so they gave only 500 rupees. But the process was very difficult, as the doctor was not signing the paper. We were in rush to come back home; my husband was running here and there. I felt like that 500 rupees were given to me like for the beggar. We told him, if you are not giving the money, we will go to the senior doctor, and then only they gave 500 rupees with so much difficulty.” (Married at 14 years, present age 17–18 years)*

Key informants claimed that women do not receive monetary benefits from the health care facility when the budget is inadequate.

Participants were also concerned about lack of health care providers and the physical structure of the facilities during their stay in hospital (power outages and mosquitoes).

Using a mixture of ‘Neem plant’ (Neem is a common tree across South Asia) and water to control itching, infections and uterine prolapse was mentioned in interviews.

“*When I started using the neem leaves and water to clean the private area it (uterine prolapse) was getting better.” (Married at 14 years, Present age 19–20 years)*

Participants considered both traditional healers and modern medicine to treat babies when they suffered from illness. A sick baby would be taken to hospital after the healer completed the healing process or had removed the affliction from the child.

*“First, he will complete his processing to get rid of ‘rahu’ (considered as inauspicious in astrology), and then we take him to the hospital.” (Married at 16 years, present age 17–18 years)*

Healers will also be consulted if problems occur during delivery:

*“My mother went to the healers for me and sacrificed chicken as told by ‘Guru’ –‘the healer’. He did the prayers for me and I delivered the baby immediately at 2 pm on Friday.” (Married at 14 years, Present age 19–20 years)*

## Discussion

Major factors acting as barriers to using health services among married adolescents in Dang district were prioritisation of domestic work, importance of first birth, mistrust towards contraceptive devices before first child, verbal abuse by health personnel, and shyness and discomfort. Lack of experience related to pregnancy and interpretation of labour pain caused them to wait to seek care. Additionally, distance and lack of transport, caste and ethnicity, and economic factors influenced the choices of health care among the participants.

Women had to perform heavy work even when pregnant. That child marriage serves to support household activities was reported in a study conducted in 15 districts from Nepal [23]. The study participants engaged in daily household work to the extent of ignoring their own health during pregnancy and after delivery. This indication of inadequate care during pregnancy is consistent with the results of a study in Pakistan [24]. However, illness that impedes household work is treated immediately, as reported in a study in India [25]. Verbal and physical violence by the husband and in-laws has also been found in other studies in Nepal among young married women [26, 27].

A study on maternal and child health in Nepal based on caste and ethnicity also revealed as our study did, that Dalits and Janajati women use antenatal health care and hospital delivery less than other groups [28].

Low decision-making ability regarding use of contraception and child bearing among married adolescents is also reported in a study from Bangladesh [29]. Our findings correspond to those reported from a multi-country study in South Asia, including Nepal, which demonstrated that child marriage is associated with not using family planning methods before first pregnancy [4]. Similar results are reported from studies among married adolescents in Bangladesh [29] and India [25]. A study in Pakistan linked child marriage with limited use of antenatal care [24].

Poor knowledge about reproductive health care and services was among the main barriers, a finding that is consistent with various studies [4, 24, 30]. Another study in Nepal found that women with better knowledge of family planning methods were more likely to prevent unintended pregnancies compared to those with less information [30]. Such lack of knowledge was also found among Bangladeshi married adolescents [29]. It is only after having one or two children that the participants come to know about and use family planning methods. This may be because their first time of contact with health care providers is during pregnancy.

Only severe pain made pregnant women to inform family members, and seek treatment, just as discrete pain during pregnancy was not considered to need medical care in a low resource setting in Vietnam [31]. This lack of knowledge and experience about the signs and symptoms of labour pain may lead to delays in seeking care that negatively influence health outcomes.

The presence of pluralistic health care in Nepal allowed participants to use multiple health care facilities [32]. A study conducted in 12 districts in Nepal indicated that adolescents use more government health care services when the services are free, accessible, and provided by skilled health care providers [33].

Young married women were motivated to seek health services by health care professionals and sometimes by older women. Older women have been found to be more motivated to seek health care compared to younger women [34, 35]. However, most participants reported that verbal abuse and judgmental attitudes from the providers, resulted in some participants feeling humiliated. Unfriendly behaviour from health care providers attending pregnant adolescents has been found in many countries, including Bangladesh [36] and Uganda [37]. A systematic review study from Africa, Asia and the Pacific reported similar results [38]. A qualitative study in Denmark found that previous experience of abuse in the health care resulted in a ‘dehumanisation’ that compelled pregnant women to delay or refrain from contacting the healthcare system [39]. The abuse experienced in health care settings by our participants appears to have had a similar effect.

As reported from Nepal previously [40] some participants had home deliveries that usually were not attended by skilled health workers. Some FGD participants supported the idea that deliveries should happen at home because they consider health risks low, which might lead women to delay seeking services until complications arise. Nasrullah et al. (2013) found that child brides in Pakistan were also likely to deliver at home [24]. Embarrassment related to sexual health found among married adolescents in a study in India might also be compatible with our study results [25].

Monetary benefits and other incentives comprise one of the approaches applied by the Nepalese government to increase the number of antenatal visits and institutional deliveries. The women are supposed to receive the benefits after the completion of all four visits [41]. In this study, the few early-married participants who knew about it found it difficult to get the money to which they were entitled.

Participants reported they had to walk a long distance or travel by bicycle or motorbike when going for antenatal care or delivery. This challenge of distance and transport was reported as a barrier to accessing health care facilities in Nepal also by Onta and colleagues [42]. Delays in reaching health care facilities may contribute to the deterioration of the health of these women [43].

### Limitations of the study

The study was conducted within four VDCs of Dang District and the findings may not be directly transferable to other rural settings or districts of Nepal. Public transport in the study area is better than in more remote areas.

## Recommendations and conclusion

Insight into young married women’s decision-making in relation to healthcare will help to design future plans and programs to improve the health status of women married as children in Nepal. Our study found more barriers than facilitators of the use of reproductive health care. More attention should thus be paid at government level to strategies regarding adolescent health care and higher priority should be given to the poor and rural settings of the country.

Women who marry and become pregnant during adolescence require more attention from health services and policy makers. A combination of the pressure to give birth early, heavy workloads, limited autonomy, little knowledge about reproductive health issues, distance to health facilities, lack of transport, and rude behaviour among health workers makes girls and young women vulnerable during pregnancy and childbirth. To train and motivate health personnel to offer quality services and treat adolescents with respect is crucial to reducing barriers to use family planning, antenatal care, delivery and post-natal care. To prevent early marriage and childbirth, the government and NGOs need to expand access to Comprehensive Sexuality Education to improve adolescent girls’ knowledge about sexual and reproductive health and their status in the families and the society.

## Abbreviations

FCHV: Female Community Health Volunteer
FGD: Focus Group Discussion
IDI: In-depth Interviews
NGOs: Non-Governmental Organisations
NHRC: Nepal Health Research Council
REK: Regional Committee for Medical and Health Research Ethics
STC: Systemic text condensation
VDCs: Village Development Committees
